# Optimizing pediatric AKI diagnosis: a patient-tailored meta-analysis of urinary TIMP-2/IGFBP7 performance across clinical scenarios and assay platforms

**DOI:** 10.3389/fped.2025.1678386

**Published:** 2025-11-17

**Authors:** Yilong Huang, Yunguang Liu, Na Lin, Guangzi Qi, Wen Shao

**Affiliations:** 1 Youjiang Medical University for Nationalities Department of Pediatrics, Affiliated Hospital of Youjiang Ethnic Medical College, Baise, China; 2 Youjiang Medical University for Nationalities, Baise, China

**Keywords:** pediatrics, meta-analysis, acute kidney injury, diagnosis, tissue inhibitor of metalloproteinases 2 (TIMP-2), insulin-like growth factor binding protein 7 (IGFBP7)

## Abstract

**Objective:**

To systematically evaluate the diagnostic value of urinary cell cycle arrest biomarkers, tissue inhibitor of metalloproteinases-2 (TIMP-2) and insulin-like growth factor binding protein 7 (IGFBP7), for the early diagnosis of acute kidney injury (AKI) in children using a meta-analysis.

**Methods:**

Nine databases were searched (inception-May 2025). Two reviewers independently screened studies, extracted data, and assessed quality (QUADAS-2). Bivariate models (STATA 18.0) generated pooled estimates.

**Results:**

15studies (1,338participants; 337 AKI cases) were included. Overall accuracy: Sensitivity 0.84 (95% CI: 0.71–0.91), Specificity 0.85 (95% CI: 0.76–0.91), AUROC 0.91. Key subgroups: High-risk Neonates: AUROC 0.96, NLR 0.13 (95% CI: 0.02–0.78), Critically Ill/Sepsis: AUROC 0.88, NLR 0.15 (95% CI: 0.04–0.64), ELISA testing: Sensitivity 0.88 (95% CI: 0.70–0.96), NLR 0.15 (95% CI: 0.05–0.43); NephroCheck® Test: AUROC 0.79; Funnel plot asymmetry suggested publication bias.

**Conclusions:**

TIMP-2/IGFBP7 show high diagnostic accuracy across pediatric settings, supporting their clinical utility.

**Systematic Review Registration:**

https://www.crd.york.ac.uk/PROSPERO/view/CRD420251089957, PROSPERO CRD420251089957.

## 1 Background

Acute kidney injury (AKI) represents a significant global health burden characterized by high mortality and poor clinical outcomes. Early detection is pivotal for effective intervention and improved prognosis (1). Diagnosing AKI in pediatric populations poses unique challenges due to ongoing physiological development and heterogeneity in clinical presentations. In response, multiple diagnostic criteria (e.g., KDIGO, pRIFLE, AKIN) have been established over recent decades by international critical care consortia to enable timely identification and mitigate mortality risks (2), underscoring the critical role of early diagnosis. Currently, the diagnosis and monitoring of acute kidney injury (AKI) primarily rely on indicators such as serum creatinine (Scr), urine output, and cystatin C (CysC). However, each of these parameters exhibits significant limitations: Scr demonstrates insufficient sensitivity and is susceptible to interference from various factors; urine output becomes less reliable in cases of non-oliguric AKI or during diuretic therapy; and CysC is prone to influences from intrinsic renal or systemic diseases, resulting in inadequate specificity (3–5). The discovery of urinary cell cycle arrest biomarkers-TIMP-2/IGFBP7-has represented a revolutionary breakthrough in AKI diagnosis. These biomarkers are rapidly up-regulated following renal injury and function by activating the p53/p21/p27 pathway, thereby inhibiting cyclin-CDK complexes and effectively mitigating cellular apoptosis or senescence (26). By reflecting cellular stress before functional impairment occurs, these biomarkers offer the potential for early clinical intervention (6). Despite their promise, robust evidence validating TIMP-2/IGFBP7 in pediatric AKI remains scarce. No comprehensive meta-analysis has yet synthesized their diagnostic performance across diverse clinical settings in children. This systematic review and meta-analysis therefore aim to: Quantify the pooled diagnostic accuracy of urinary TIMP-2/IGFBP7 for pediatric AKI; Evaluate their performance in key clinical subgroups (e.g., postoperative, critically ill, neonatal); Identify gaps to guide clinical implementation and future research.

## 2 Materials and methods

### 2.1 Literature search

This meta-analysis adhered to the Preferred Reporting Items for Systematic Reviews and Meta-Analyses (PRISMA) and the PRISMA-Diagnostic Test Accuracy (PRISMA-DTA) guidelines (7). The protocol was prospectively registered in the International Prospective Register of Systematic Reviews (PROSPERO; CRD420251089957).

A systematic search strategy was executed across nine databases: English Databases: PubMed, Embase, Cochrane Library, Web of Science, Google Scholar, Chinese Databases: China National Knowledge Infrastructure (CNKI), Wanfang Data, VIP Information (VIP), SinoMed (CBM).Search Terms included: Child, Infant, Acute Kidney Injury, Kidney Injuries, Tissue Inhibitor of Metalloproteinase-2, TIMP-2, insulin-like growth factor binding protein-related protein 1, IGFBP-7 (with synonyms and MeSH terms adapted per database).

No restrictions were applied regarding language, region, or publication date. Animal studies were excluded. The search spanned from database inception to May 2025. Additional studies were identified by manually screening reference lists of included articles.

### 2.2 Inclusion criteria

Studies were eligible if they met all of the following: Participants aged <18 years; Diagnostic test accuracy studies evaluating urinary TIMP-2 and/or IGFBP7 for early AKI detection; Sufficient data to derive or calculate true positive (TP), false positive (FP), false negative (FN), and true negative (TN) values; The diagnosis of AKI was based on internationally recognized criteria, including pRIFLE, AKIN, and KDIGO, or subtype-specific definitions such as CI-AKI or Sepsis-AKI, with quantifiable declines in renal function (e.g., serum creatinine elevation or decreased urine output).

### 2.3 Exclusion criteria

Studies were excluded if they: Enrolled participants aged ≥18 years; Were non-primary research (conference abstracts, guidelines, letters, reviews, case reports); Represented duplicate publications; Provided insufficient data to construct a 2 × 2 contingency table.

### 2.4 Quality assessment and data extraction

Two independent reviewers with expertise in nephrology and meta-analysis: Screened titles/abstracts and assessed full texts against eligibility criteria; Extracted data using a standardized form, including: First author, publication year, country, study design, population characteristics, age range, biomarker(s) assessed, sample size, assay method, sampling timepoint, AKI definition, TP/FP/FN/TN; Evaluated methodological quality using the Quality Assessment of Diagnostic Accuracy Studies 2 (QUADAS-2) tool in RevMan 5.4 (8, 9). Discrepancies were resolved through consensus discussion or consultation with a third reviewer.

### 2.5 Subgroup definitions and rationale

All subgroup analyses were prospectively specified in the PROSPERO protocol.

Surgery/Invasive Procedures-studies of cardiopulmonary bypass, liver transplant, contrast nephropathy, etc.

Critically Ill/Sepsis-pediatric ICU cohorts with sepsis-related AKI or multiorgan failure.

High-Risk Neonates-preterm or very-low-birth-weight infants at risk of AKI (e.g., asphyxia, PDA treatment).

### 2.6 Statistical analysis

Analyses were performed using RevMan 5.4 (The Cochrane Collaboration) and STATA 18.0 (StataCorp LP). Heterogeneity Assessment: Quantified via Cochran's *Q* test and I²statistic. Fixed-effect models were applied if heterogeneity was negligible (I² < 50% and *P* ≥ 0.1); Random-effects models were used for significant heterogeneity (I² ≥ 50% and *P* < 0.1). Diagnostic Accuracy Metrics: Bivariate generalized linear mixed models (*via* the midas command in STATA) generated: Pooled sensitivity, specificity, positive/negative likelihood ratios (PLR/NLR) Diagnostic odds ratios (DOR) Summary receiver operating characteristic (SROC) curves with area under the curve (AUC) Publication Bias:Assessed via funnel plot asymmetry.

## 3 Results

### 3.1 Literature selection

**Figure 1:**
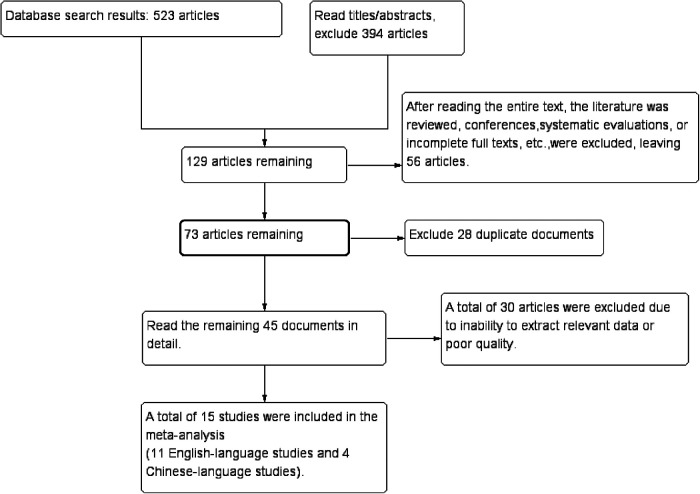
Screening process of articles.

Initial database searches identified 523 records. After screening titles/abstracts, 394 records were excluded. Full-text review excluded 56 articles (reviews, conference abstracts, or inaccessible full texts), 28 duplicates, and 30 studies with incomplete data or low quality. Fifteen studies (10–24) met eligibility criteria (Figure 1).

### 3.2 Study characteristics

The 15 included studies (10–24) enrolled 1,338 participants, comprising 337 AKI cases and 1,001 non-AKI controls. Key study characteristics and extracted data are summarized in Tables 1, 2.

**Table 1:** General characteristics of included studies.

Author	Year	Country	Patients	Study Design	Population Characteristics	Age Range	Diagnostic Criteria
			AKI/Total				
Ismail et al. (10)	2024	Egypt	52/72	Case-control	PICU	Median 0.5 years	pRIFLE
Ramírez et al. (11)	2022	USA	19/36	Prospective cohort	Post-cardiac surgery	Neonates	AKIN
Ganda et al. (12)	2022	Indonesia	40/113	Prospective cohort	Pediatric sepsis	1 month–18 years	KDIGO
Selim et al. (13)	2018	Egypt	36/60	Prospective cohort	PICU	1 month–16 years	pRIFLE
Chen et al. (14)	2020	China	9/226	Prospective cohort	NICU	Neonates	KDIGO
Sun et al. (15)	2022	China	20/137	Prospective cohort	Children receiving intravascular contrast media	0–18 years	CI-AKI definition
Meersch et al. (16)	2014	Germany	12/51	Prospective cohort	Post-cardiac surgery	0–18 years	pRIFLE
Raffaella et al. (17)	2025	Italy	3/26	Prospective cohort	Preterm infants	Neonates	KDIGO
Gist et al. (18)	2017	USA	31/94	Prospective cohort	VLBW infants receiving indomethacin therapy	≤1 year	KDIGO
Sina et al. (19)	2019	Germany	3/32	Prospective cohort	VLBW infants receiving indomethacin therapy	Preterm neonates	KDIGO
Fuhrman et al. (20)	2020	USA	6/16	Prospective cohort	Children undergoing first liver transplantation	Median 8.5 years	KDIGO
Dai et al. (21)	2019	China	11/144	Prospective cohort	PICU	>1 month	KDIGO
Gao et al. (22)	2022	China	34/106	Prospective cohort	Pediatric sepsis	1–12 years	KDIGO
Xiao et al. (23)	2018	China	52/174	Prospective cohort	Pediatric sepsis	1–12 years	KDIGO
Yan et al. (24)	2021	China	9/51	Prospective cohort	Neonates with severe asphyxia	Neonates	KDIGO

CI-AKI, Contrast-Induced Acute Kidney Injury (34): A decline in renal function occurring within 72 h after intravascular contrast exposure, after excluding alternative etiologies, defined as: Absolute serum creatinine (SCr) increase > 44 μmol/L (0.5 mg/dL), or Relative SCr increase ≥ 25% from baseline.

**Table 2:** Characteristics and biomarker assessment of included studies.

Author	Year	TP	FP	FN	TN	Biomarkers	Assay Methods
Ismail et al. (10)	2024	29	5	23	15	TIMP-2*IGFBP7	ELISA
Ramírez et al. (11)	2022	13	2	6	15	TIMP-2*IGFBP7	NephroCheck® Test
Ganda et al. (12)	2022	30	18	10	55	IGFBP-7	ELISA
Selim et al. (13)	2018	36	0	0	24	TIMP-2	ELISA
Chen et al. (14)	2020	8	107	1	110	TIMP-2*IGFBP7	ELISA
Sun et al. (15)	2022	16	22	4	95	TIMP-2*IGFBP7	ELISA
Meersch et al. (16)	2014	10	9	2	30	TIMP-2*IGFBP7	NephroCheck® Test
Raffaella et al. (17)	2025	1	0	2	23	TIMP-2*IGFBP7	NephroCheck® Test
Gist et al. (18)	2017	18	20	13	43	TIMP-2*IGFBP7	NephroCheck® Test
Sina et al. (19)	2019	3	0	0	29	TIMP-2*IGFBP7	NephroCheck® Test
Fuhrman et al. (20)	2020	5	1	1	9	TIMP-2*IGFBP7	NephroCheck® Test
Dai et al. (21)	2019	8	28	3	105	IGFBP-7	ELISA
Gao et al. (22)	2022	23	9	11	63	TIMP-2	ELISA
Xiao et al. (23)	2018	51	18	1	104	TIMP-2*IGFBP7	ELISA
Yan et al. (24)	2021	9	5	0	37	TIMP-2*IGFBP7	ELISA

Data in this table reflect original study-level findings, summarizing diagnostic 2 × 2 contingency data from primary studies. Due to heterogeneity across studies-including variations in: Population characteristics (e.g., age distribution, comorbidities), Biomarker sampling timepoints, Diagnostic threshold criteria-certain metrics may exhibit inter-study variability.

### 3.3 Risk of bias assessment

**Figure 2:**
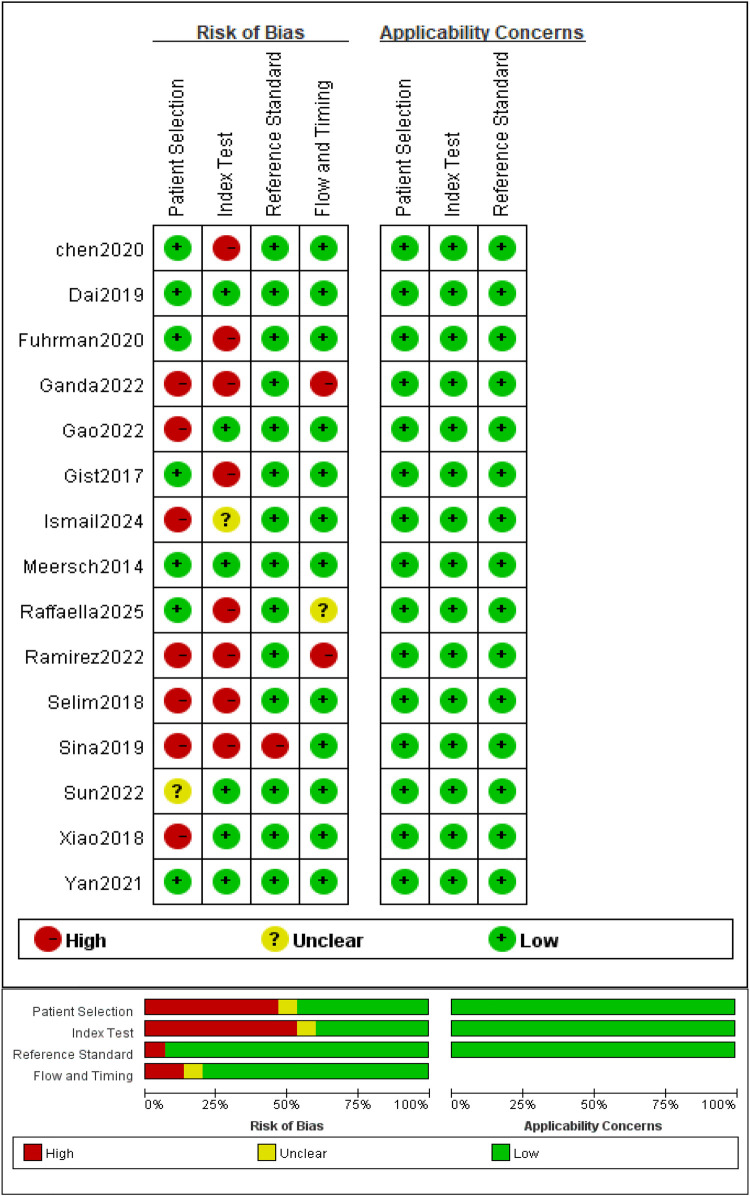
Methodological quality summary of risk of bias and applicability concerns using QUADAS-2.

Methodological quality was evaluated using the QUADAS-2 tool. Results are presented as a risk-of-bias summary table and proportion plot (Figure 2).

### 3.4 Meta-analysis of diagnostic accuracy

**Figure 3:**
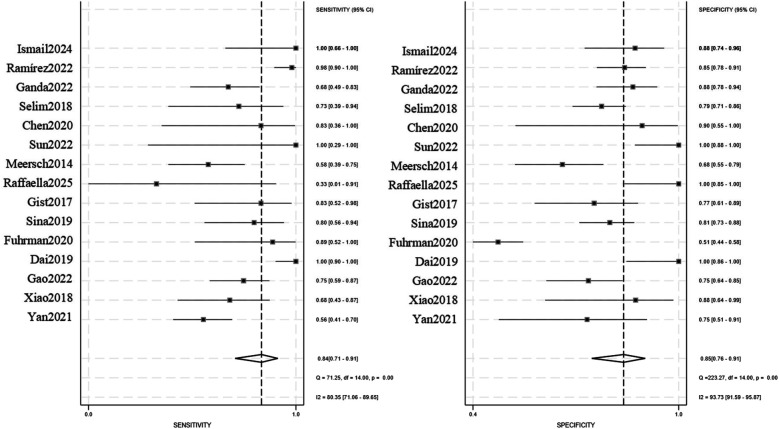
Sensitivity/specificity forest plot (TIMP-2/IGFBP7).

**Figure 4:**
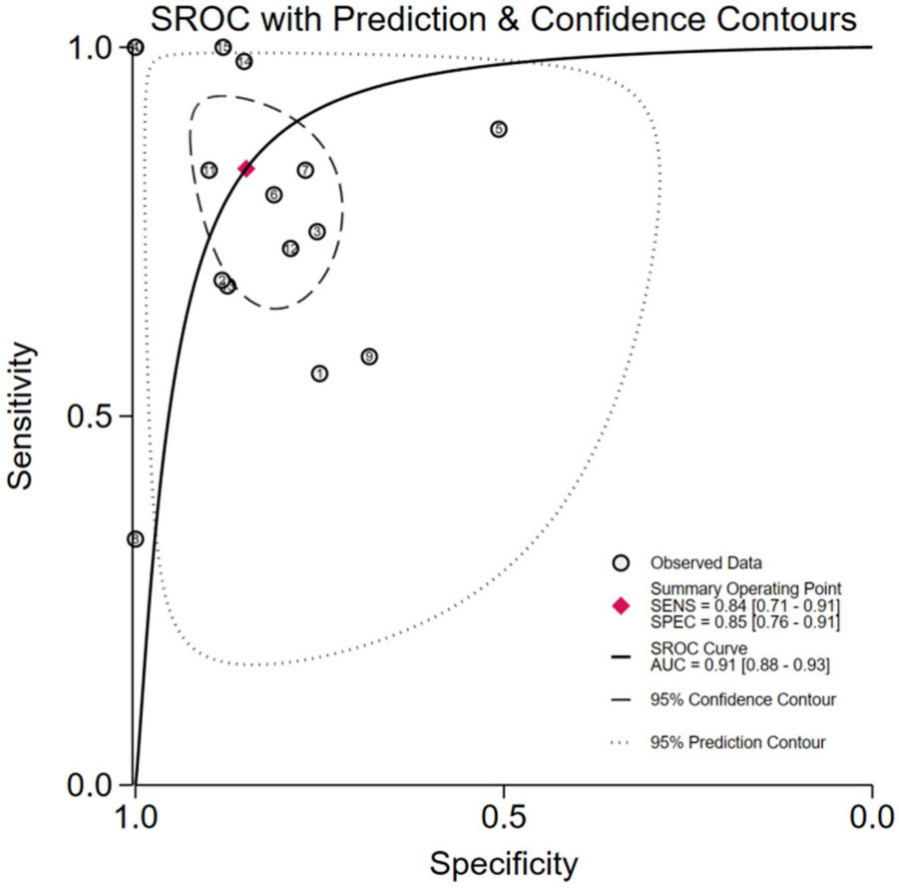
SROC curve (TIMP-2/IGFBP7).

The pooled analysis demonstrated significant diagnostic utility of urinary TIMP-2/IGFBP7 (combined or individual) for pediatric AKI: Sensitivity: 0.84 (95% CI: 0.71–0.91), Specificity: 0.85 (95% CI: 0.76–0.91), AUROC): 0.91 (95% CI: 0.88–0.93), Positive Likelihood Ratio (PLR): 5.6, Negative Likelihood Ratio (NLR): 0.19, Diagnostic Odds Ratio (DOR): 29, Substantial heterogeneity was observed: Sensitivity: *I*^2^ = 80.35%, Cochran's *Q* = 71.25 (*P* < 0.001), Specificity: *I*^2^ = 93.73%, Cochran's *Q* = 223.27 (*P* < 0.001), A random-effects model was therefore applied. Subgroup analyses were conducted to explore heterogeneity sources (Figures 3, 4).

### 3.5 Publication bias

**Figure 5:**
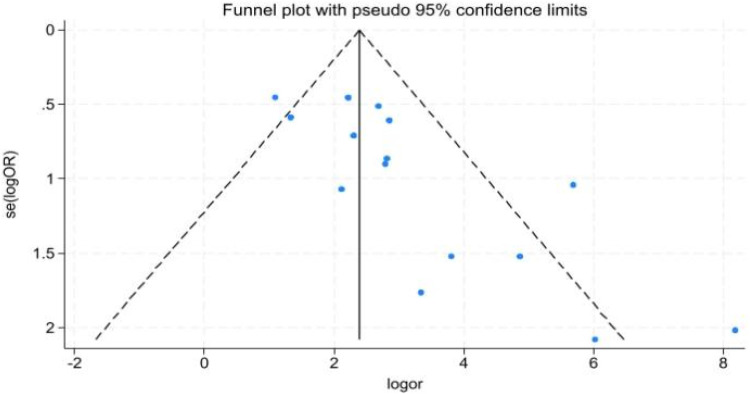
Publication bias funnel plot.

Funnel plot asymmetry (Figure 5) suggested potential publication bias.

### 3.6 Subgroup analyses

(Summarized in Table 3).

**Table 3:** Subgroup analysis of TIMP-2/IGFBP7 diagnostic performance statistics for pediatric AKI.

Participants Included in Analysis	AKI/Non-AKI	SEN (95% CI)	SPE (95% CI)	PLR (95% CI)	NLR (95% CI)	DOR (95% CI)	AUROC (95% CI)	Cochran's Q statistic	*I*^2^ statistic with 95% confidence interval (95% CI)
all studies	337/1,001	0.84 (0.71, 0.91)	0.85 (0.76, 0.91)	5.6 (3.3, 9.4)	0.19 (0.10, 0.37)	29 (10, 83)	0.91 (0.88, 0.93)	8.160 (*p* = 0.008)	75% (46%, 100%)
Surgery/Invasive Procedures	88/246	0.73 (0.59, 0.83)	0.78 (0.71, 0.84)	3.3 (2.3, 4.9)	0.35 (0.22, 0.56)	10 (4, 21)	0.83 (0.79, 0.86)	0.088 (*p* = 0.479)	0% (0%, 100%)
High-risk Neonates	24/311	0.87 (0.46, 0.98)	0.97 (0.38, 1.00)	30.4 (0.7, 1,319.8)	0.13 (0.02, 0.78)	232 (6, 8,941)	0.96 (0.94, 0.97)	9.804 (*p* = 0.004)	80% (56%, 100%)
Critically Ill/Sepsis	225/444	0.87 (0.59, 0.97)	0.83 (0.76, 0.89)	5.2 (3.1, 8.9)	0.15 (0.04, 0.64)	35 (5, 240)	0.88 (0.85, 0.91)	9.172 (*p* = 0.005)	78% (53%, 100%)
Combined Biomarker Testing	216/699	0.82 (0.66, 0.91)	0.84 (0.73, 0.91)	5.3 (2.9, 9.7)	0.21 (0.10, 0.43)	25 (8, 76)	0.9 (0.87, 0.93)	9.325 (*p* = 0.005)	79% (54%, 100%)
Single Biomarker Testing	121/302	0.86 (0.51, 0.97)	0.89 (0.64, 0.97)	8.0 (1.7, 37.6)	0.16 (0.03, 0.81)	51 (2, 1,064)	0.94 (0.92, 0.96)	0.135 (*p* = 0.467)	0 (0%, 100%)
ELISA Testing	263/820	0.88 (0.70, 0.96)	0.82 (0.72, 0.89)	4.8 (2.8, 8.4)	0.15 (0.05, 0.43)	32 (7, 142)	0.9 (0.87, 0.93)	8.994 (*p* = 0.006)	78% (52%, 100%)
ELISA for Combined Biomarkers Only	142/518	0.9 (0.65, 0.98)	0.77 (0.63, 0.87)	3.9 (2.1, 7.3)	0.13 (0.03, 0.58)	30 (4, 207)	0.88 (0.85, 0.91)	7.287 (*p* = 0.013)	73% (39%, 100%)
NephroCheck® Test	74/181	0.71 (0.54, 0.83)	0.92 (0.73, 0.98)	8.5 (2.2, 32.1)	0.32 (0.19, 0.54)	27 (5, 143)	0.79 (0.75, 0.82)	3.471 (*p* = 0.088)	42% (0%, 100%)

#### 3.6.1 Clinical subgroups

A. Surgery/Invasive Procedures (5 studies; 88 AKI cases, 246 non-AKI controls): Sensitivity: 0.73 (95% CI: 0.59–0.83), Specificity: 0.78 (95% CI: 0.71–0.84), PLR: 3.3 (95% CI: 2.3–4.9), NLR: 0.35 (95% CI: 0.22–0.56), DOR: 10 (95% CI: 4–21), AUROC: 0.83 (95% CI: 0.79–0.86), Heterogeneity: *I*^2^ = 0% (*Q* = 0.088, *P* = 0.479).

B. High-risk Neonates (4 studies; 24 AKI cases, 311 non-AKI controls): Sensitivity: 0.87 (95% CI: 0.46–0.98), Specificity: 0.97 (95% CI: 0.38–1.00), PLR: 30.4 (95% CI: 0.7–1,319.8), NLR: 0.13 (95% CI: 0.02–0.78), DOR: 232 (95% CI: 6–8,941), AUROC: 0.96 (95% CI: 0.94–0.97), Heterogeneity: *I*^2^ = 80% (*Q* = 9.804, *P* = 0.004).

C. Critically Ill/Sepsis (6 studies; 225 AKI cases, 444 non-AKI controls): Sensitivity: 0.87 (95% CI: 0.59–0.97), Specificity: 0.83 (95% CI: 0.76–0.89), PLR: 5.2 (95% CI: 3.1–8.9), NLR: 0.15 (95% CI: 0.04–0.64), DOR: 35 (95% CI: 5–240), AUROC: 0.88 (95% CI: 0.85–0.91), Heterogeneity: *I*^2^ = 78% (*Q* = 9.172, *P* = 0.005).

#### 3.6.2 Analytical strategies

A. Combined Biomarker Testing (11 studies; 216 AKI cases, 699 non-AKI controls): Sensitivity: 0.82 (95% CI: 0.66–0.91), Specificity: 0.84 (95% CI: 0.73–0.91), PLR: 5.3 (95% CI: 2.9–9.7), NLR: 0.21 (95% CI: 0.10–0.43), DOR: 25 (95% CI: 8–76), AUROC: 0.90 (95% CI: 0.87–0.93), Heterogeneity: *I*^2^ = 79% (*Q* = 9.325, *P* = 0.005).

B. Single Biomarker Testing (4 studies; 121 AKI cases, 302 non-AKI controls): Sensitivity: 0.86 (95% CI: 0.51–0.97), Specificity: 0.89 (95% CI: 0.64–0.97), PLR: 8.0 (95% CI: 1.7–37.6), NLR: 0.16 (95% CI: 0.03–0.81), DOR: 51 (95% CI: 2–1,064), AUROC: 0.94 (95% CI: 0.92–0.96), Heterogeneity: *I*^2^ = 0% (*Q* = 0.135, *P* = 0.467).

#### 3.6.3 Assay methods

A. ELISA Testing (9 studies; 263 AKI cases, 820 non-AKI controls): Sensitivity: 0.88 (95% CI: 0.70–0.96), Specificity: 0.82 (95% CI: 0.72–0.89), PLR: 4.8 (95% CI: 2.8–8.4), NLR: 0.15 (95% CI: 0.05–0.43), DOR: 32 (95% CI: 7–142), AUROC: 0.90 (95% CI: 0.87–0.93), Heterogeneity: *I*^2^ = 78% (*Q* = 8.994, *P* = 0.006).

Subanalysis: ELISA for Combined Biomarkers Only (5 studies; 142 AKI cases, 518 non-AKI controls): Sensitivity: 0.90 (95% CI: 0.65–0.98), Specificity: 0.77 (95% CI: 0.63–0.87), PLR: 3.9 (95% CI: 2.1–7.3), NLR: 0.13 (95% CI: 0.03–0.58), DOR: 30 (95% CI: 4–207), AUROC: 0.88 (95% CI: 0.85–0.91), Heterogeneity: *I*^2^ = 73% (*Q* = 7.287, *P* = 0.013).

B. NephroCheck® Test (6 studies; 74 AKI cases, 181 non-AKI controls): Sensitivity: 0.71 (95% CI: 0.54–0.83), Specificity: 0.92 (95% CI: 0.73–0.98), PLR: 8.5 (95% CI: 2.2–32.1), NLR: 0.32 (95% CI: 0.19–0.54), DOR: 27 (95% CI: 5–143), AUROC: 0.79 (95% CI: 0.75–0.82), Heterogeneity: *I*^2^ = 42% (*Q* = 3.471, *P* = 0.088).

## 4 Discussion

Acute kidney injury (AKI) in children is a common critical illness that poses a serious threat to the health and well-being of pediatric patients. It is characterized by complex and diverse etiologies, poor prognosis, and the early diagnosis and treatment of AKI are of critical importance for improving patient outcomes (25). Currently, the diagnosis of AKI in children primarily relies on biomarkers such as Scr and CysC for estimating glomerular filtration rate (GFR). Although these indicators offer certain clinical utility, they are susceptible to various interfering factors, leading to limitations such as delayed diagnosis and suboptimal sensitivity. Moreover, while studies have explored the diagnostic potential of other biomarkers like β2-microglobulin (β2-MG), evidence supporting their use as well-established diagnostic tools remains insufficient, and they have not gained broad clinical acceptance. Urinary cell cycle arrest markers (TIMP-2/IGFBP7), first validated as AKI biomarkers by Kashani et al. (26) and later FDA-approved, demonstrate significant diagnostic potential.

In recent years, with the deepening of research into the pathophysiological mechanisms of AKI, urinary cell cycle arrest markers (TIMP-2/IGFBP7) have gradually become a research hotspot. Therefore, this study conducted a preliminary assessment of the diagnostic value of TIMP-2 and IGFBP7 in urine through a meta-analysis, aiming to provide clinicians with a more reliable approach for the early diagnosis of pediatric AKI through evidence-based medicine.

In this meta-analysis, a total of 15 studies (10–24) were included, with an overall pooled sensitivity of *I*^2^ = 80.35%, and specificity *I*^2^ = 93.73%, indicating high heterogeneity among the studies. Subgroup analyses were conducted based on different population characteristics, detection methods, and single or combined detection. However, the results of this meta-analysis indicate that, regardless of the approach, urinary cell cycle arrest markers (TIMP-2/IGFBP7) have significant diagnostic value for the occurrence of pediatric AKI.

Previous studies have shown that surgery and imaging are common risk factors for pediatric AKI. Acute kidney injury is particularly common in infants following cardiac surgery (27). Subgroup analysis of invasive procedures such as surgery and imaging revealed that in this specific population of children undergoing surgical or invasive procedures, the AUROC was 0.83, the diagnostic odds ratio was 10 (95% CI: 4–21), and the sensitivity was 0.73 (95% CI: 0.59–0.83), which is lower than its superior performance in the overall pediatric AKI population (AUROC = 0.91). However, the meta-analysis results showed high consistency (*I*^2^ = 0%), indicating that the application of this marker in this specific, high-risk population remains reliable and stable, providing clinical physicians with a basis for diagnosing AKI.

Due to the unique characteristics of renal development and pathophysiology in children, the incidence of AKI varies across different age groups. Xu et al. (28) reported in a large multicenter study that the incidence of AKI in infants and toddlers in China is several times higher than that in adolescents. Additionally, other studies have suggested that younger age may also be a contributing factor to higher mortality rates (29). Given this, we conducted a subgroup analysis of the high-risk neonatal cohort. The AUROC reached 0.96, and the negative likelihood ratio was 0.13 (95% CI: 0.02–0.78), the lowest among all subgroups, indicating its potential to effectively avoid unnecessary interventions. However, due to the limited sample size, the positive likelihood ratio exhibited an extremely wide confidence interval (95% CI: 0.7–1,319.8), reflecting high uncertainty in the estimate. Therefore, these results must be interpreted with caution. Nonetheless, the findings suggest the potential value of this biomarker in neonatal medicine. These preliminary results urgently require validation in prospective, large-scale neonatal cohort studies.

Critically ill and sepsis patients treated in the PICU are also a high-risk group for AKI, and sepsis is a strong associated factor for AKI in PICU patients, with AKI significantly increasing their risk of death (30). The results of this meta-analysis for critically ill children and children with sepsis treated in the PICU showed *Q* = 9.172, *P* = 0.005, and *I*^2^ = 78%, indicating high heterogeneity and significant differences between studies. Considering that AKI may occur postoperatively in PICU patients and is included in the critically ill group, along with the complexity of sepsis etiology (bacterial/viral/fungal infections, varying degrees of multi-organ failure) (31), which may be the cause of the high heterogeneity. Nevertheless, the sensitivity of TIMP-2 and IGFBP7 for diagnosing AKI in critically ill and sepsis patients remains as high as 0.87, and the negative likelihood ratio is 0.15 (95% CI: 0.04–0.64), indicating a considerable ability to rule out AKI. This has important implications for guiding clinical practice and facilitating early renal protection measures.

Given the diverse testing methodologies across the included studies, it is also crucial to compare the performance of different diagnostic platforms. However, some hospitals or research institutions may not have access to the NephroCheck® Test for detecting [TIMP-2] × [IGFBP7], so ELISA serves as an alternative method. To clarify the potential differences between the two detection methods, a meta-subgroup analysis was conducted, using ELISA to detect the TIMP-2 and IGFBP7 markers in the subgroup. The meta-analysis results showed a high sensitivity of 0.88 (95% CI: 0.70–0.96), which may be attributed to the mature enzyme-substrate colorimetric system and continuous technological iterations of ELISA, significantly enhancing its accuracy. Additionally, ELISA offers notable advantages such as relative ease of operation, batch testing capability, and low cost (32) making it particularly suitable for institutions and hospitals with limited resources. In this meta-analysis, the specificity was 0.82 (95% CI: 0.72–0.89) and the positive likelihood ratio was 4.8 (95% CI: 2.8–8.4), suggesting that it may be more suitable for early screening of AKI rather than definitive diagnosis while the negative likelihood ratio of 0.15 (95% CI: 0.05–0.43) indicates a high negative predictive value. In emergency departments or general wards, a negative result from the rapid ELISA method can effectively and safely rule out AKI, but this requires further evidence-based medical research to confirm. The NephroCheck® Test also demonstrated good diagnostic performance, but its AUROC was only 0.79. Since the NephroCheck® Test is a combined assay for [TIMP-2] × [IGFBP7], it excludes single marker testing. Therefore, by excluding single marker studies, the ELISA subgroup was optimized. In the subgroup of the combined [TIMP-2] × [IGFBP7] biomarker detection using ELISA technology, the sensitivity exceeded 0.9 (95% CI: 0.65–0.98), the negative likelihood ratio was as low as 0.13 (95% CI: 0.03–0.58), and the AUROC was 0.88, and its diagnostic performance remains superior to the NephroCheck® Test. In 2014, the FDA approved the use of ASTUTE140® for the [TIMP-2] × [IGFBP7] detection of the NephroCheck® Test, and the package insert specified that the test is only intended for patients aged 21 years and older (33), aligning with its suboptimal performance in pediatric cohorts (AUROC = 0.79).

Following the identification of these two biomarkers as diagnostic markers for AKI, most tests are performed as combined AKI tests, while single-marker tests are relatively rare. Therefore, in the subgroup comparisons of this meta-analysis, the diagnostic value of combined testing of urinary cell cycle arrest biomarkers (TIMP-2/IGFBP7) vs. single-marker testing for pediatric AKI was systematically evaluated. The results showed that although both groups demonstrated high diagnostic performance, there were differences: combined testing was significantly superior to single-marker testing in terms of a narrower confidence interval for sensitivity and a smaller confidence interval range for the diagnostic odds ratio. Although single-marker testing had higher values for some indicators, its clinical applicability was limited due to small sample sizes and highly unstable results. This aligns with Professor Kashani's assertion that the two markers appear to be more predictive when used together (26).

The studies included in this meta-analysis had varying sample sizes, and most were single-center studies, with funnel plots showing asymmetry, indicating potential publication bias. Prior to conducting the meta-analysis, a comprehensive literature search was performed across major global databases to minimize the risk of omissions and reduce potential bias. However, during the meta-analysis, there may have been some bias and heterogeneity due to potential confounding effects from mixed statistics between single-marker testing and combined testing in severe sepsis and septicemia. Moreover, the lack of uniformity in AKI diagnostic criteria (pRIFLE, AKIN, KDIGO) across the included studies constitutes an important source of clinical heterogeneity. However, due to the small sample size and data sparsity within the relevant subgroups, it was not feasible to perform statistically meaningful comparisons. Out of methodological rigor, this meta-analysis refrained from discussing potential outcome differences arising from the use of different AKI diagnostic criteria. This limitation also highlights a clear direction for future investigations aimed specifically at comparing the performance of these diagnostic standards. Nevertheless, the core conclusion is clear: urinary cell cycle arrest biomarkers (TIMP-2/IGFBP7) demonstrate consistent high diagnostic performance across all subgroups (different population characteristics, single/dual biomarker testing, and different testing technologies), confirming their universal diagnostic value for pediatric AKI. However, establishing more appropriate diagnostic criteria for children may further assist clinicians in assessing disease severity. Additionally, determining suitable diagnostic thresholds for the combined detection of [TIMP-2] × [IGFBP7] in urine for pediatric AKI could overcome the limitations of the current adult thresholds in the NephroCheck® Test, thereby enhancing its applicability in pediatrics. Based on the above evidence, we recommend the implementation of a structured precision medicine pathway termed “High-Risk Screening—Biomarker Verification—Risk-Stratified Management” in clinical settings. This approach involves first identifying high-risk children using established clinical risk factors (e.g., cardiac surgery, sepsis, or PICU admission). Depending on available medical resources, urinary [TIMP-2] × [IGFBP7] levels should then be measured using either ELISA or the NephroCheck® Test assay to facilitate objective risk stratification. Management should be triaged based on the results: low-risk children can avoid unnecessary interventions, while high-risk children should receive early nephroprotective therapy. This pathway is highly feasible and adaptable to healthcare institutions with varying resource levels. Nonetheless, large-scale, multi-center clinical studies remain necessary to further validate the diagnostic value of these biomarkers in pediatric AKI and promote their integration into routine clinical practice.

## Data Availability

The original contributions presented in the study are included in the article/Supplementary Material, further inquiries can be directed to the corresponding author.
